# Scientific Publication Patterns of Systematic Reviews on Psychosocial Interventions Improving Well-being: Bibliometric Analysis

**DOI:** 10.2196/41456

**Published:** 2022-11-11

**Authors:** Ivanna Shubina

**Affiliations:** 1 Liberal Arts Department American University of the Middle East Egaila Kuwait

**Keywords:** psychosocial intervention, well-being, systematic review, bibliometric analysis, bibliometrics, scientific research, medical research, publication, publish, citation, scientometrics, mental health

## Abstract

**Background:**

Despite numerous empirical studies and systematic reviews conducted on the effectiveness of interventions improving psychological well-being, there is no holistic overview of published systematic reviews in this field.

**Objective:**

This bibliometric study explored the scientific patterns of the effectiveness of different psychosocial interventions improving well-being among various categories of individuals with mental and physical diseases, to synthesize well-being intervention studies, and to suggest gaps and further studies in this emerging field.

**Methods:**

The bibliometric analysis included identifying the most productive authors, institutions, and countries; most explored fields and subjects of study; most active journals and publishers; and performing citation analysis and analyzing publication trends between 2014 and 2022. We focused on data retrieved from known databases, and the study was conducted with a proven bibliometric approach.

**Results:**

In total, 156 studies were found concerning the research domains and retrieved using LENS software from high-ranking databases (Crossref, Microsoft Academic, PubMed, and Core). These papers were written in English by 100 authors from 24 countries, among which, the leading country was the United Kingdom. Descriptive characteristics of the publications involved an increased number of publications in 2017 (n=35) and 2019 (n=34) and a decreased number in 2021 (n=4). The top 2 leading authors by citation score are James Thomas (3 papers and 260 citations) and Chris Dickens (3 papers and 182 citations). However, the most cited study had 592 citations. *BMJ Open* (n=6 articles) is the leading journal in the field of medicine; *Clinical Psychology Review* (n=5), in psychology; and *Frontiers in Psychology*, in psychological intervention (n=5) and psychology (n=5). The top 2 publishers were Wiley (n=28) and Elsevier (n=25).

**Conclusions:**

This study indicates an overall interest in the declared domains within the last decade. Our findings primarily indicate that psychosocial interventions (PIs) were evaluated as being effective in managing mental and physical problems and enhancing well-being. Cognitive behavioral therapy was assessed as being effective in treating anxiety, psychoeducation in relapse prevention, and gratitude interventions in improving overall health, and the mindfulness approach had a positive impact on decreasing distress and depression. Moreover, all these intervention types resulted in an overall increase in an individuals’ well-being and resilience. Integrating social and cultural factors while considering individual differences increases the efficiency of PIs. Furthermore, PIs were evaluated as being effective in managing symptoms of eating disorders, dementia, and cancer. Our findings could help provide researchers an overview of the publication trends on research domains of focus for further studies, since it shows current findings and potential research needs in these fields, and would also benefit practitioners working on increasing their own and their patients' well-being.

## Introduction

### Background

The effectiveness of available interventions for improving well-being is one of the major research questions that scientists and practitioners are exploring nowadays. Psychoeducational interventions were evaluated as effective in increasing compliance and preventing relapse among family carers of individuals with psychosis [1]. The gratitude interventions have a significant impact on individuals’ physical and mental health [2]. Despite small numbers and low-quality data, some of them supported the efficiency of acceptance and commitment therapy in parenting of children with long-term conditions, seizure control in epilepsy, psychological flexibility, and self-management [3]. CBT was considered effective in the treatment of anxiety among individuals with asthma rather than treatment of the illness itself [4]. Carolan et al [5] stated no significant difference between studies using cognitive behavioral therapy and those using other psychological interventions.

A systematic review on mindfulness approach highlighted positive personal experiences and professional benefits among participants, such as reinforcement of their clinical skills and attitudes [6]. Mindfulness meditation resulted in positive outcomes in relation to distress, burnout, and depression among health care professionals [7], overall increase in staff well-being and resilience [8], along with a decrease in distress and blood pressure [9]. Evidence presented in a systematic review by Alsubaie et al [10] suggests increased effectiveness of mindfulness-based cognitive therapy. Duarte et al [11] identified and evaluated economic evidence for mindfulness meditation in improving mental health and stated inadequate data to generalize the findings.

The social and cultural factors need to be incorporated into the design and implementation of interventions to increase their efficiency [12]. In addition, possessing skills that allow attitude change, adjustment of the content to the target group, and matching the gender and ethnicity of the person delivering the intervention and the recipient are considered significant factors [13]. However, individual differences should be considered an influential determinant in a psychological intervention’s efficiency [14]. A multicomponent psychosocial intervention (PI) was evaluated as being effective in improving cognitive functioning, social interaction, and well-being [15] and in decreasing pain [16] among patients with dementia. A systematic review by Shen et al [17] supports the effectiveness of PIs combined with family-based models, education, supportive services for caregivers, and abuse of older individuals. Another study suggested that physical activity is positively correlated but sedentary behaviors are negatively associated with psychosocial well-being in early childhood [18]. Delivering positive experiences, destigmatization, and use of a person-centered approach are recommended for effectively treating dementia [19]. However, a systematic review on the effectiveness of psychological interventions supporting patients with cancer in increasing their life quality stated insufficient data to claim its efficiency [20].

A combination of internal and external factors enables carers of patients with a cancer diagnosis to experience positive emotions [21]. A “Schwartz Rounds” environment [22] and interventions enhancing work engagement, including personal resource–building, job resource–building, leadership training, and health promotion [23], were all evaluated as being effective in providing support to health care staff with managing emotional challenges at work and improving their well-being. Graham et al [3] focused on exploring the life quality among health care professionals helping patients with eating disorders, while Narzisi and Simons [24] analyzed evidence of interventions preventing obesity among children. It has been stated that evidence- and theory-based interventions are more effective in promoting healthy eating habits [25]. A study on the holistic treatment of patients with obesity reported positive effects on awareness, health behavior, and physical activity and led to a decrease in drinking and an increase in well-being and self-efficacy [26]. The negative impact of stigma on psychological well-being among patients with ED was reported in a mixed methods systematic review by O’Connor et al [27].

A systematic review by Attwood et al [28] appraised the interventions for health care professionals to improve their negative attitudes toward personality disorders. Vereenooghe et al [29] investigated the effectiveness of psychological and pharmacological interventions for mental health problems among individuals with severe intellectual disabilities. Merkouris et al [30] recognized the significant predictors (eg, being employed, no gambling debt, and personality traits), unclear predictors (eg, treatment goal), and nonsignificant predictors (eg, education, income, anxiety, substance use, etc) for disordered gambling.

The multilevel parenting intervention program showed its positive impact at each level, resulting in an improvement of well-being among children, parents, and families [31]. The idea of using PIs with adoptive parents [32] and evidence-based parenting interventions [33] are effective for enhancing children’s well-being. Peters et al [34] suggested that the areas related to the parents’ perception of infants’ mental health are important. It has been shown that parental interventions decrease maternal depressive symptoms [35] and can be positively associated with educational, health, and well-being effects as well as economic benefits [36].

To sum up, many recent studies suggested that psychological, social, digital, and other interventions are effective approaches in increasing an individuals’ well-being. However, there is no overview of available systematic reviews and meta-analyses, which synthesized the analyzed qualitative and quantitative studies in the indicated research domains. This bibliometric study is aimed to analyze the objectives and synthesize the findings of identified systematic reviews on the effectiveness of different PIs directed on increasing well-being among children, adults, and professional staff experiencing a physical or a mental illness.

### Purpose of the Study and Research Questions

The primary purpose of this study is to explore scientific publication patterns in systematic reviews encompassing research domains of PIs and well-being. This study also aims to reveal the contribution of scientific knowledge by highlighting the contributions, gaps, and direct potential further studies. Based on the research objectives and scope, the following research questions have been formulated: (1) What are the descriptive characteristics of publication results? (2) Who are the most productive authors or coauthors, and what are their institutions and fields of study? What are the citation results of those authors? (3) Which organizations, countries, sources, and publishers contribute to the research area? (4) What are the results of keyword analysis of the publications?

## Methods

### Bibliometric Study

The bibliometric study provides the opportunity for researchers to investigate existing scientific patterns, trends, and associations in searched domains and interrelated fields over identified publication data. For bibliometric analysis to be successful, it requires a structured database with the appropriate data that will allow the researchers to answer the aforementioned research questions [37-43].

Bibliometrics uses statistical methods to analyze scholarly publications in a wide spectrum such as peer-reviewed journal articles, e-books, conference proceedings, periodicals, reviews, and reports. The bibliometric study, as a method, offers a range of tools for analyzing both, empirical studies and literature reviews [39-45]. In this study, the author employed descriptive publication results, author or coauthor, institutions and country productivity, source and publisher productivity, and most common MeSH (Medical Subject Headings) and keyword analysis [40,43,44].

### Data Collection and Extraction

An efficient bibliometric study requires a well-structured database to analyze available and relevant publication data. The main bibliometric databases available for this paper are Crossref (n=156 papers), Microsoft Academic (n=151), PubMed (n=156), Core (n=147), and PubMed Central (n=79). All databases have their citation count categories. Thus, publication data were retrieved from the aforementioned reputed databases with the following search strategy. The search time frame was 2014 to 2022, since there were no relevant publications indicated before 2014. We included only systematic reviews (n=156) with the following search query: *positive AND (psychology AND (interventions AND well-being))*.

The abovementioned search criteria were conducted, and the data were retrieved as plain .txt and excel .csv file formats for further analysis. The Microsoft Excel and Lens platform (version 7.4) software with the “bibliometrix” package was used for descriptive and bibliometric data analysis.

Bibliometric data were obtained by first identifying all extracted articles in the Lens databases [46]. To ensure accuracy, results from a comprehensive Lens search of all papers published from 2014 to 2022 were cross-referenced and matched between the highly ranked databases (Crossref, Microsoft Academic, PubMed, PubMed Central, and Core). The discrepancy between the total numbers in each database was checked manually. No duplication or missing studies were identified; thus, accurate matching was accomplished. The resultant list from Lens software was exported into a Microsoft Excel spreadsheet, and the visualized data were saved as images.

Validation of the search query was based on reviewing the top 156 cited documents about PIs and well-being to ensure that they fit within the scope of the research field. This approach was adopted to eliminate false positive results by excluding documents focusing on the impact of other approaches or any document irrelevant to the explored subject.

## Results

### Publication Profile and Descriptive Publication Results

A total of 156 relevant publications for the identified research domain were retrieved from the Lens database. The papers were written in English by 100 corresponding authors or coauthors from 24 different countries, where the leading country is the United Kingdom, followed by Australia and the United States. Descriptive characteristics of the publications show an increase in the number of studies in 2017 (n=35) and 2019 (n=34) and a decrease in 2021 (n=4). The top 4 fields to which the published papers belong are medicine (n=84), psychological intervention (n=80), psychology (n=62), and clinical psychology (n=43).

### Distribution of Publications by Date

**Figure 1:**
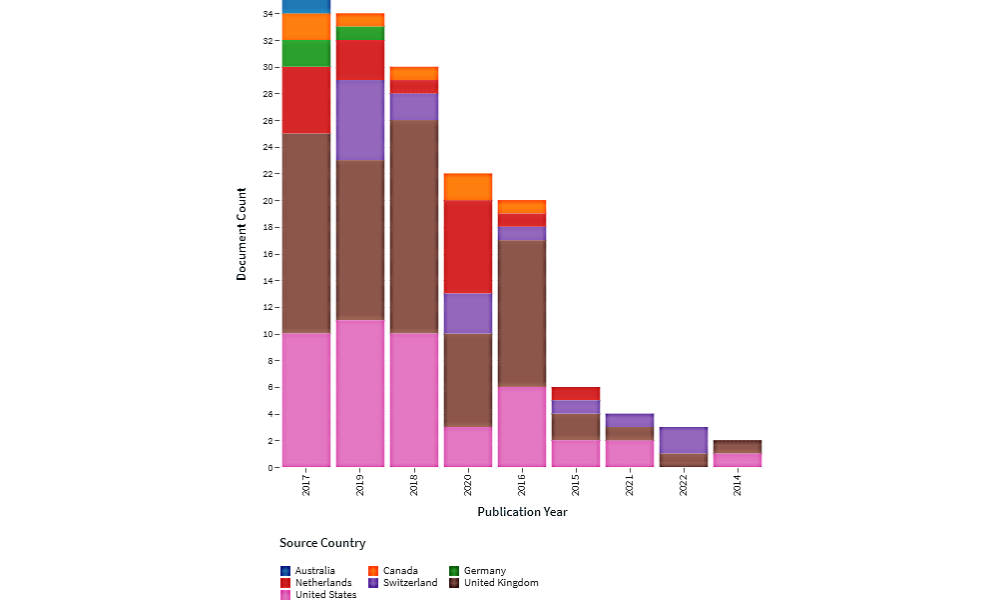
Publication records by date of publishing and most productive country (2014-2022).

Figure 1 shows the dynamics of publishing papers about PIs and well-being in different countries in 2014-2022.

Records include 2 papers in 2014, which then significantly increased in 2016 (n=20) and 2017 (n=35). In 2018, this number slightly decreased (n=30), again increased in 2019 (n=34), and decreased in 2021 (n=4) and 2022 (n=3). In 2017 (one of the most productive years), the most productive countries were the United Kingdom (n=15) and the United States (n=10), followed by the Netherlands (n=5). In 2019, the United States was leading in publishing papers within the studied domain (n=11), followed by the United Kingdom (n=12), Switzerland (n=6), and the Netherlands (n=3). The United Kingdom and the United States were productive in publishing papers on PIs and well-being during 2014-2022. Canada was one of the most productive countries in 2017 (n=2) and 2020 (n=2), with a total of 7 papers published during the studied period.

### Field and Subject of Study

This research domain is categorized by the top 10 major fields of study (out of 100 in total) with the number of published studies. According to our results, most published papers in PI and well-being are related to the following fields of study: medicine (n=84), psychological intervention (n=80), psychology (n=62), and clinical psychology (n=43). Obviously, some of the papers were qualified to a few fields of study (see Multimedia Appendix 1). The most often explored subjects among published papers on PIs and well-being are psychiatry and mental health (n=45), general medicine (n=26), clinical psychology (n=20), general psychology (n=13), developmental and educational psychology (n=12), and health informatics (n=12) (see Multimedia Appendix 1).

### Most Productive Authors or Coauthors and Institutions

Table 1 presents the top 10 most productive authors by the total number of cited papers; it also presents the number of publications and author productivity measured by the average citations per published paper. The top 4 leading authors are as follows: James Thomas with 3 published papers and 260 citations, followed by Chris Dickens with 3 papers and 182 citations, Brendon Stubbs with 2 papers and 72 citations, and Catherine Meads with 2 papers and 53 citations. Other authors wrote 2-4 papers and received 20 citations.

According to the obtained results, the top 10 cited articles in the studied domain, Sanders et al [31] was the leading paper and has the highest citation score (592/6847, 8.6% counts) (see Multimedia Appendix 1).

**Figure 2:**
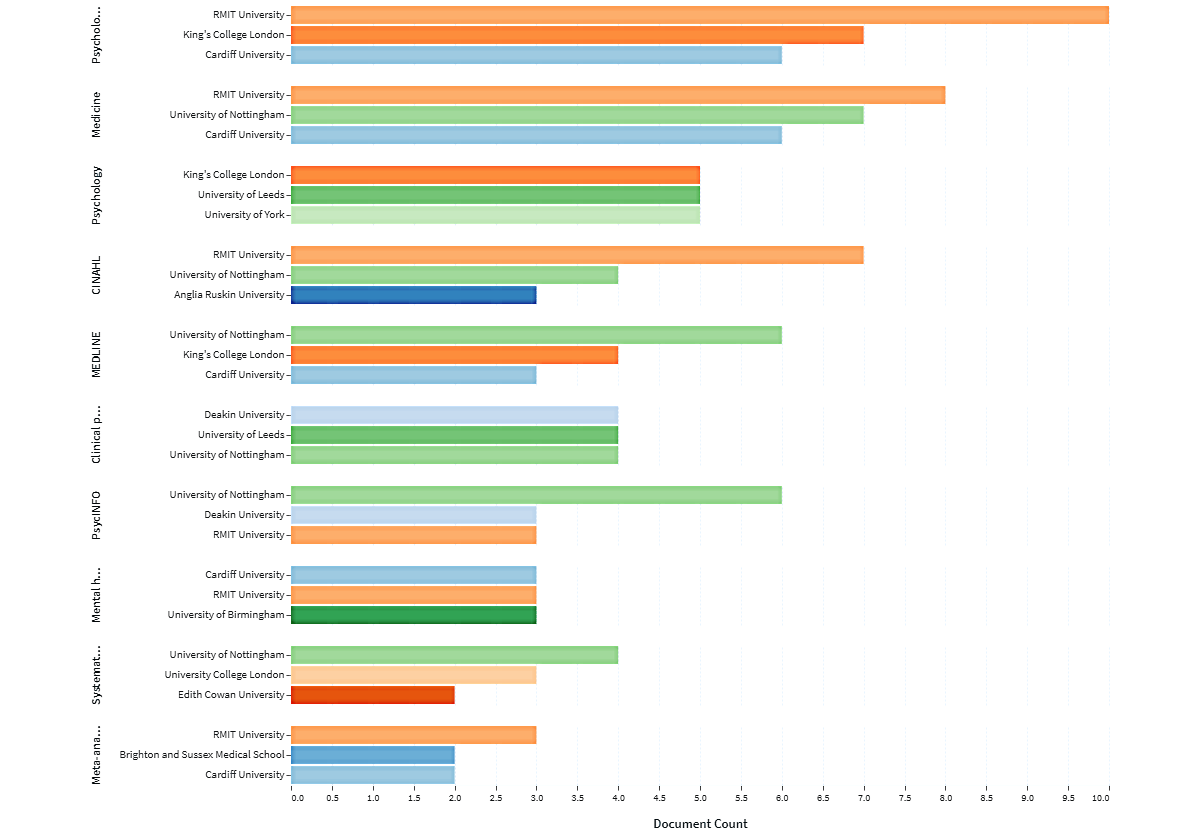
Publication records by institutions and field of study. RMIT: Royal Melbourne Institute of Technology.

Figure 2 shows the most productive (top 10) institutions by field of study and year of publication. The Royal Melbourne Institute of Technology is one of the leading institutions in publishing papers in psychological intervention (n=10), medicine (n=8), and mental health (n=3) and in CINAHL (n=7) and PsycINFO (n=3); King’s College London, in the field of psychological intervention (n=7) and psychology (n=5) and in MEDLINE (n=4); and Cardiff University, in psychological intervention (n=6), medicine (n=6), and mental health (n=3) and in MEDLINE (n=3). Another leading institution is the University of Nottingham in the fields of medicine (n=7), clinical psychology (n=4), and mental health (n=3) and in MEDLINE (n=6) and PsycINFO (n=6).

**Table 1:** Top 10 authors by document count, sum, and average citing of scholarly works.

Author	Sum of cited scholarly works, n	Publications, n	Productivity (average citation per paper), mean
James Thomas	260	3	87
Chris Dickens	182	3	61
Brendon Stubbs	143	2	72
Catherine Meads	105	2	53
Chris Bonell	85	3	28
Ruth Garside	85	3	28
Colette Joy Browning	85	2	42
Claudio Di Lorito	71	4	17
Andrew Thompson	64	2	32
Alessandro Bosco	60	3	20

### Most Productive Journals and Publishers

**Figure 3:**
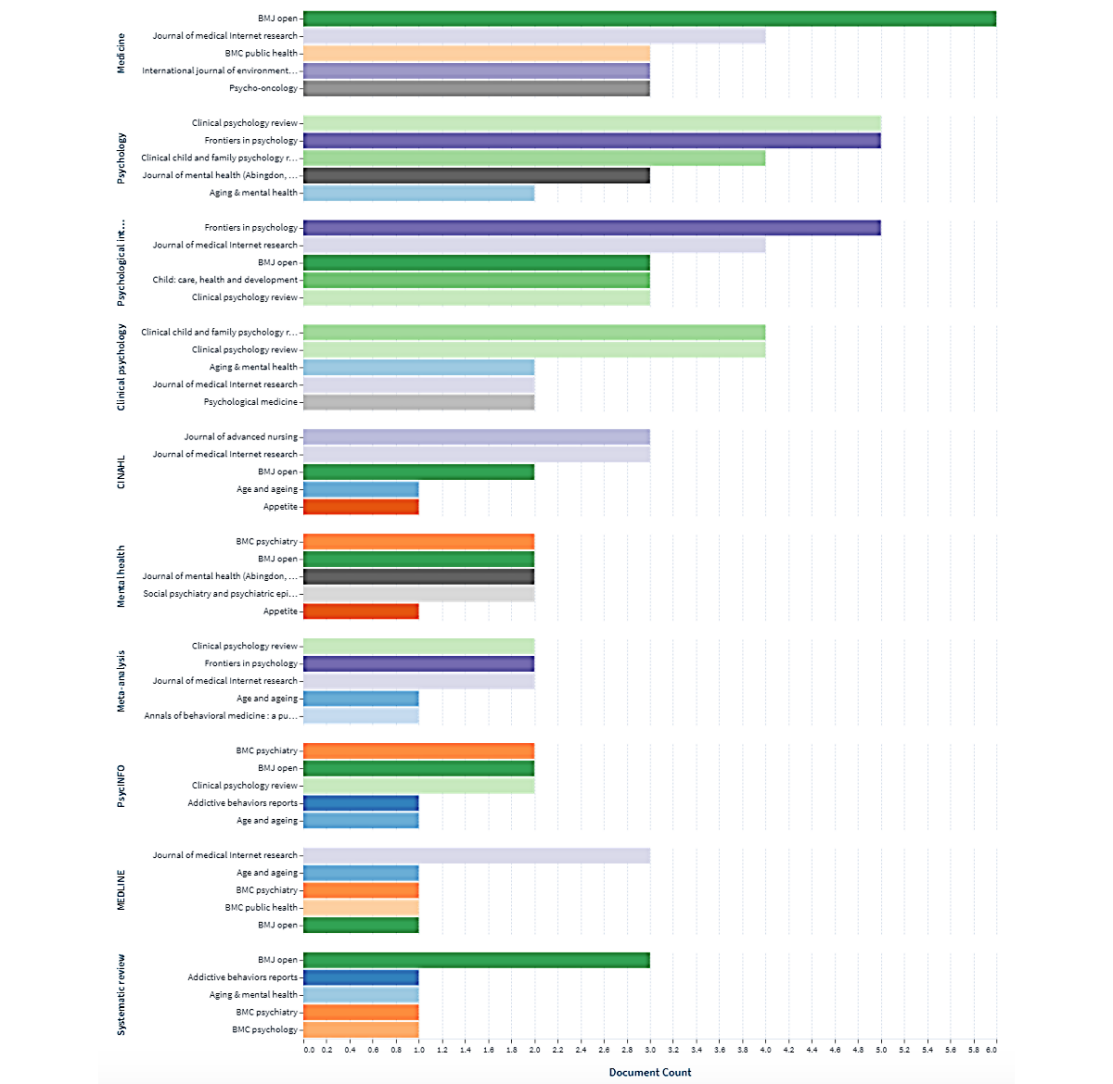
Most productive journals by publication count and field of study.

Figure 3 shows the top 5 productive journals belonging to each of the top 10 fields of study. According to our results, *BMJ Open* (n=6) and *Journal of Medical Internet Research* (n=4) are leading in the field of medicine. In the field of psychology, the most productive journal is *Clinical Psychology Review* (n=5), while *Frontiers in Psychology* is one of leading journals in psychology (n=5) and psychological intervention (n=5). The field of clinical psychology was represented by *Clinical Psychology Review* (n=4) and *Journal of Clinical Child & Adolescent Psychology* (n=4). *BMC Psychiatry*, *BMJ Open*, *Journal of Mental Health*, and *Social Psychiatry and Psychiatric Epidemiology* are the most productive journals in the field of mental health with 2 published papers each.

Table 2 provides data on the most productive publishers by document count, sum, and average citing of scholarly works. According to our results, the top 3 publishers in the domain of PI and well-being are Wiley (n=28), Elsevier (n=25), and BioMed Central (n=15). However, the most cited papers were published by Elsevier (n=2560). The 3 least productive publishers in these domains are Multidisciplinary Digital Publishing Institute (8 published papers and 105 citations in total), Frontiers Media SA (6 published papers and 101 citations in total), and SAGE Publications (7 published papers and 168 citations in total).

According to our results, the most productive journals are *International Journal of Environmental Research and Public Health* (7 papers; Multidisciplinary Digital Publishing Institute), followed by *BMJ Open* (6 papers; PubMed), and *Frontiers in Psychology* (6 papers; Frontiers Media SA). *Child: Care, Health and Development* and *Journal of Advanced Nursing* are the most productive journal (4 papers; Wiley). *BMC Psychiatry* and *BMC Public Health* (3 papers; BioMed Central) are leading in publishing papers, while *Clinical Psychology Review* (6 papers; Elsevier) is the most productive journal (see Multimedia Appendix 1).

Table 3 shows the top 10 productive countries by document count, average author count, and the sum and average of scholarly citations.

According to our results, the top 3 productive countries by document count are the United Kingdom (n=126), Australia (n=30), and the United States (n=14). The top 3 countries by average author or coauthor count are Australia (n=78), Germany (n=65), and the United States (n=63). The 3 leading countries by the sum of citations are the United Kingdom (n=5700), Australia (n=2350), and the United States (n=880). The most productive countries by average citation score are Switzerland (n=130), Australia (n=78), and Germany (n=65). New Zealand (n=15) is the least productive country with 4 publications and 60 citations in total.

**Table 2:** Top 10 publishers by document count, sum, and average citing of scholarly works.

Publisher	Publications, n	Sum of cited scholarly works, n	Productivity (average citation per papers), mean
Wiley	28	710	25
Elsevier	25	2560	102
BioMed Central	15	602	40
Multidisciplinary Digital Publishing Institute	8	105	13
SAGE Publications	7	168	24
BMJ Publishing Group	6	169	28
Frontiers Media SA	6	101	17
Oxford University Press	6	215	35
JMIR Publications	5	444	89
Academic Press	4	203	51

**Table 3:** Top 10 countries by document count, author average count, and the sum and average of scholarly citations.

Country	Publication, n	Average authors count, n	Sum of scholarly citations, n	Productivity (average citation per paper), mean
United Kingdom	126	45	5700	45
Australia	30	78	2350	78
United States	14	63	880	62
Germany	6	65	390	65
New Zealand	4	15	60	15
Switzerland	3	13	390	130
Spain	3	58	175	58
Ireland	3	57	170	57
Netherlands	3	27	80	27
China	2	40	80	40

### Keyword Analysis and MeSH

MeSH terms are assigned to PubMed entries by the National Library of Medicine at the National Institutes of Health. This analysis reveals the frequency of the MeSH terms used in analyzed publications. Table 4 shows the most frequently used MeSH terms in the publications associated with PIs for well-being. The first column represents MeSH terms and the second one shows article counts.

Multimedia Appendix 2 illustrates two different word cloud–based data distributions. The left side shows a word cloud of keywords by document count, whereas the right side represents keywords by the sum of citations. According to the obtained results, the top 3 cited keywords are “meta-analysis” (n=1740, average citation score=66.9), “well-being” (n=1218, average citation score=76.1), and “public health” (n=897, average citation score=112.1). The least cited keywords are “intervention” (n=183, average citation score=22.8) and “behavioral change” (n=207, average citation score=51.7).

**Table 4:** Most relevant top 10 MeSH (Medical Subject Headings) terms according to PubMed.

MeSH terms	Article count, n
Humans	132
Adult	28
Females	28
Male	23
Child	21
Adolescent	21
Quality of life	16
Aged	14
Mental health	13
Qualitative research	11

## Discussion

### Principal Findings

This bibliometric analysis was conducted to investigate existing scientific patterns, trends, and associations in extracted systematic reviews on the effectiveness of PIs aimed to improve well-being among children, adults, and professional staff experiencing physical or mental illness. The text analysis highlighted the most frequent subject categories and fields along with the keywords and MeSH terms.

By analyzing the results of the data set regarding PIs improving the well-being of individuals with various psychological and physical problems published in well-known databases, the rapid increase in research interest in this subject was clearly indicated. The trend sharply increased in recent years between 2016 and 2020. This growth in interest could reflect the decrease in psychological and physical well-being among health care professionals, adults, and youths; therefore, the need for effective PIs supporting individuals in improving their overall well-being has become crucial.

The wide range of available PIs (including the psychological, psychoeducational, and parental interventions and the mindfulness approach) were recognized as effective in managing mental health and physical problems and in increasing overall well-being [1,3,6]. Cognitive behavioral therapy was evaluated as effective in the treatment of anxiety [4]; psychoeducational interventions, in relapse prevention [1]; and gratitude interventions, in the improvement of overall health [2].

Mindfulness training had a positive impact on decreasing burnout, distress, and depression among health care staff [7], improving their clinical skills and attitudes [6] and increasing their resilience overall [8]. Integrating social and cultural factors [12], considering individual differences [14] and skills for change and adjustment to the group target [13], increases the efficiency of interventions, especially for cognitive functioning, social interaction, and well-being [15]. Work engagement, personal resources, and leadership skills increase the probability of experiencing positive emotions [21,22] and managing emotional challenges at work more effectively [23]. PIs that help increase self-awareness and self-efficacy are effective in managing eating disorders and improve an individuals’ well-being overall [24,26,47]. PIs for parents were evaluated as effective for adopted children [32], parents of infants [34], and mothers with maternal depressive symptoms [35], resulting in an improvement of well-being among children, parents, and whole families [31].

Even though there is increasing interest in the research domains explored in this study, no publication using a bibliometric approach has been found to analyze the PIs for improving individuals' well-being. Therefore, the uniqueness of this study could itself be considered its strength. This study reveals scientific patterns and future research gaps to academics and practitioners. Text analysis also highlighted and supported popular subject areas to clarify the research scope and directions.

One of the limitations of this study is that the number of relevant studies made precise content analysis more challenging; however, this was beyond the scope and aim of this study. Although the various bibliometric analysis methods exist in the literature, the scope and size of the research led authors to concentrate on more specific analysis such as descriptive statistics with this data set of studies from 2014 to 2022.

### Gaps and Future Scope

According to our findings, the aforementioned research domains are prevalent, and an upward trend in interest can be seen for publication records since 2016. Besides, the majority of the subject category records were found in the fields of medicine, PIs, psychology, and clinical psychology. Further research would be conducted with various aspects of bibliometric analysis including systematic reviews, meta-analysis, and empirical studies. Moreover, the studies assessed here indicate the importance of further explorations and analyses to develop appropriate and feasible PIs for the improvement of well-being and managing mental and physical health problems.

Focus has to be directed on how to optimize intervention design to improve an individual’s well-being in the long term [1,48] and how to encourage and measure the maintenance of psychological, social, and environmental changes [47,49].

Further research should develop criteria for the evaluation of interventions’ effectiveness in improving psychological well-being [50,51], and assess individual experiences across a range of PI [14] and treatment–response associations in particular [18].

More research on intervention type, intensity, duration, and follow-up measurement [28] is required for a more precise evaluation of the effectiveness of PI for patients with cancer [20] and those with dementia [15].

Further research is required to provide recommendations about the effectiveness of interventions with adoptive parents [32] and parents of infants to improve their mental well-being in later life [34].

More studies are needed to explore the benefits of group work interventions [52] and to design work interventions appropriate to individual conditions and expectations [23]. Exploration of the benefits of group interventions, characteristics of professionals, and impact of motivational strategies on intervention delivery and outcomes would be beneficial [53].

Future research on PIs should focus on the needs of older patients [54] and connections between intervention activities and ultimate change in behaviors related to abuse of older individuals [17].

There is a need for methodological standards in testing the mechanisms of mindfulness-based treatment [10], their cost‐effectiveness for mental health conditions [11], and individual readiness for mindfulness-based interventions [6].

### Conclusions

This bibliometric study aimed to explore and analyze the scientific patterns and relations of scholarly publications on the effectiveness of PIs for improving well-being among individuals with psychological or physical conditions. Therefore, various forms of bibliometric methods have been employed, and findings were illustrated with a data visualization approach. The bibliometric analysis was conducted on 156 systematic reviews published in highly ranked databases between 2014 and 2022.

Our results present the most frequent subject and field categories, popular keywords, productive authors, countries and institutions, active journals, and publishers within the chosen domain. This bibliometric study revealed the patterns of publication and critical areas in the data set and provides insights and research directions for academics, practitioners, and readers who wish to collaborate in this domain for the future.

A total of 156 relevant publications were retrieved and analyzed from highly ranked databases (eg, PubMed, Crossref, and Microsoft Academic). Among the top 3 leading authors with respect to the number of citations per paper are the following: James Thomas with 3 published papers and 260 citations, followed by Chris Dickens with 3 papers and 182 citations, and Brendon Stubbs with 2 papers and 72 citations. However, the most cited paper was that of Sanders et al [31] with 592 out of 6847 (8.6%) citations; this was followed by Johnson et al [55] with a citation record of 561 (8.2%), followed by Strauss et al [56] with a citation count of 335 (5%). According to the obtained results, the top 2 cited keywords are “well-being” (n=1218, average citation score=76.1) and “public health” (n=897, average citation score=112.1). The least cited keywords are “intervention” (n=183, average citation score=22.8) and “behavioral change” (n=207, average citation score=51.7).

According to the analyzed data, *BMJ Open* (n=6) and *Journal of Medical Internet Research* (n=4) are leading in the field of medicine; *Clinical Psychology Review* (n=5), in psychology; and *Frontiers in Psychology*, in psychology (n=5) and psychological intervention (n=5). Clinical psychology is represented by *Clinical Psychology Review* (n=4) and *Journal of Clinical Child & Adolescent Psychology* (n=4). The top 3 publishers in the domain of PIs and well-being are Wiley (n=28), Elsevier (n=25), and BioMed Central (n=15). However, the most cited papers were published by Elsevier (n=2560).

The data discussed here indicate the significance of further explorations in this field to adjust the certain positive psychology interventions to the individual and situational factors to increase their effectiveness in improving overall well-being among individuals with mental and physical problems.

## Appendix

Multimedia Appendix 1 Top 10 fields of study by publication count, study characteristics, and their distribution by publisher.

Multimedia Appendix 2 Keywords by document count and sum citing scholarly work count (limited to 50).

## Abbreviations

MeSH: Medical Subject Headings
PI: psychosocial intervention
