# Supplementary material for: Scientific Publication Patterns of Systematic Reviews on Psychosocial Interventions Improving Well-being: Bibliometric Analysis
Source: Interact J Med Res. 2022 Nov 11;11(2):e41456. doi: 10.2196/41456 (PMC9700239; doi:10.2196/41456)

**Table S1.** Top 10 fields of study by publications count (2014-2021).

| Field of study | Publications (n) |
| --- | --- |
| Medicine | 84 |
| Psychological intervention | 80 |
| Psychology | 62 |
| Clinical psychology | 43 |
| CINAHL | 38 |
| MEDLINE | 33 |
| PsycINFO | 33 |
| Mental health | 26 |
| Population | 24 |
| Intervention (counseling) | 22 |


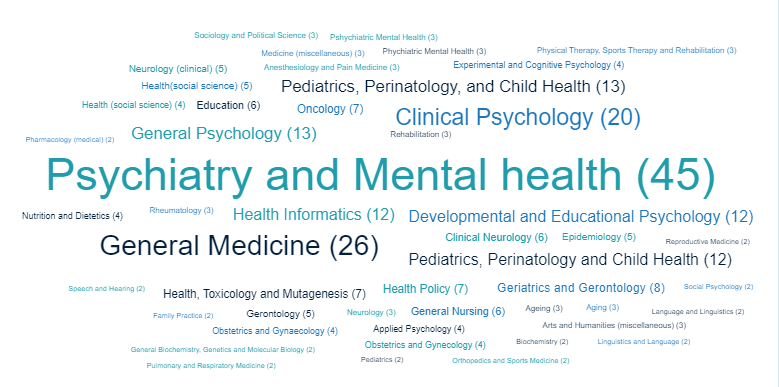


**Figure S1.** Word cloud of the top subjects by publication count.

**Table S2.** Characteristics of the studies.


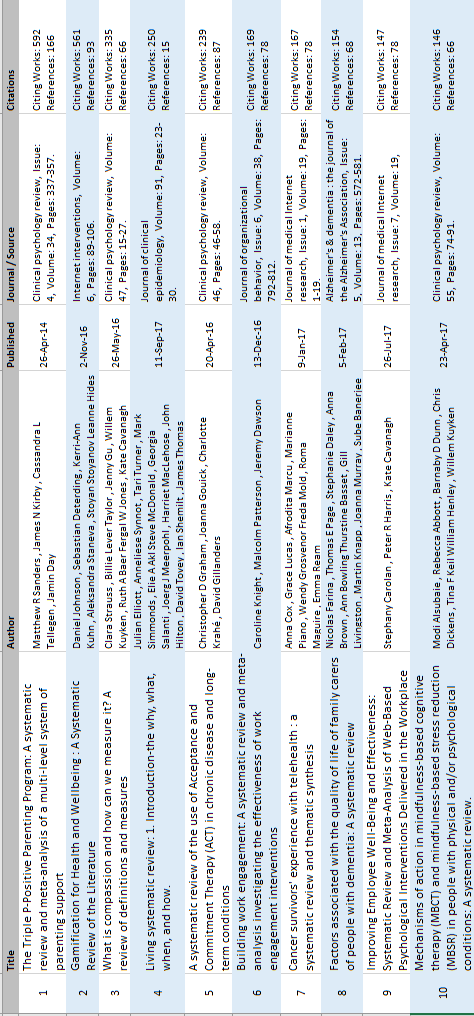


**Figure S2.** Subject areas by publisher.


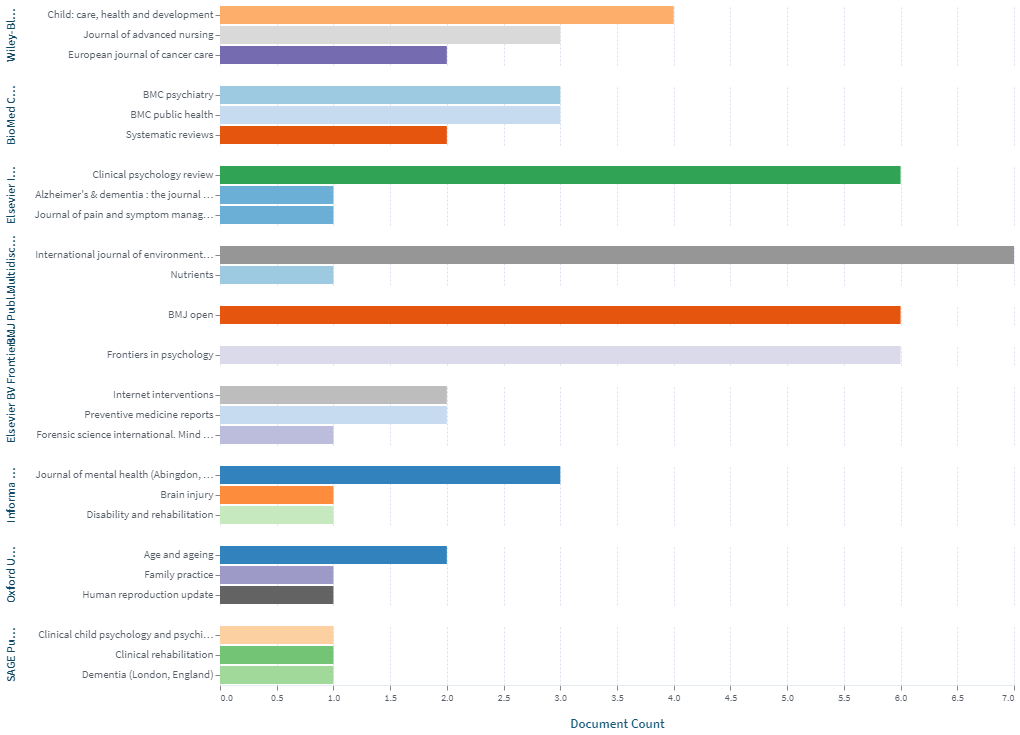

Supplement: Multimedia Appendix 1 [file ijmr_v11i2e41456_app1.docx]
