# Supplementary figures and images for: Scientific Publication Patterns of Systematic Reviews on Psychosocial Interventions Improving Well-being: Bibliometric Analysis
Source: Interact J Med Res. 2022 Nov 11;11(2):e41456. doi: 10.2196/41456 (PMC9700239; doi:10.2196/41456)

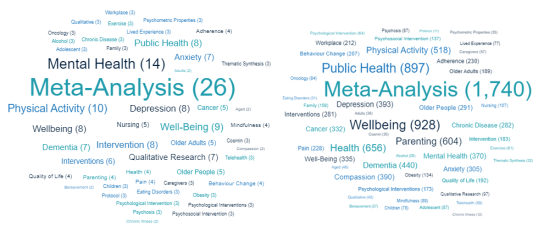

Supplement: Multimedia Appendix 2 [file ijmr_v11i2e41456_app2.png]
